# A quick and reliable image-based AI algorithm for evaluating cellular senescence of gastric organoids

**DOI:** 10.20892/j.issn.2095-3941.2023.0099

**Published:** 2023-06-30

**Authors:** Ruixin Yang, Yutong Du, Wingyan Kwan, Ranlin Yan, Qimeng Shi, Lu Zang, Zhenggang Zhu, Jianming Zhang, Chen Li, Yingyan Yu

**Affiliations:** 1 Department of General Surgery of Ruijin Hospital, Shanghai Institute of Digestive Surgery, Shanghai Key Laboratory for Gastric Neoplasms, Shanghai Jiaotong University School of Medicine, Shanghai 200025, China; 2 Institute of Translational Medicine, Zhangjiang Institute for Advanced Study, Shanghai Jiao Tong University, Shanghai 201210, China

**Keywords:** Gastric cancer, organoids, cellular senescence, senescence-associated β-galactosidase, artificial intelligence

## Abstract

**Objective:**

Organoids are a powerful tool with broad application prospects in biomedicine. Notably, they provide alternatives to animal models for testing potential drugs before clinical trials. However, the number of passages for which organoids maintain cellular vitality *ex vivo* remains unclear.

**Methods:**

Herein, we constructed 55 gastric organoids from 35 individuals, serially passaged the organoids, and captured microscopic images for phenotypic evaluation. Senescence-associated β-galactosidase (SA-β-Gal), cell diameter in suspension, and gene expression reflecting cell cycle regulation were examined. The YOLOv3 object detection algorithm integrated with a convolutional block attention module (CBAM) was used to evaluate organoid vitality.

**Results:**

SA-β-Gal staining intensity; single-cell diameter; and expression of *p15*, *p16*, *p21*, *CCNA2*, *CCNE2*, and *LMNB1* reflected the progression of aging in organoids during passaging. The CBAM-YOLOv3 algorithm precisely evaluated aging organoids on the basis of organoid average diameter, organoid number, and number × diameter, and the findings positively correlated with SA-β-Gal staining and single-cell diameter. Organoids derived from normal gastric mucosa had limited passaging ability (passages 1–5), before aging, whereas tumor organoids showed unlimited passaging potential for more than 45 passages (511 days) without showing clear senescence.

**Conclusions:**

Given the lack of indicators for evaluating organoid growth status, we established a reliable approach for integrated analysis of phenotypic parameters that uses an artificial intelligence algorithm to indicate organoid vitality. This method enables precise evaluation of organoid status in biomedical studies and monitoring of living biobanks.

## Introduction

At the end of 2022, new legislation was signed by President Joe Biden regarding the approval of new drugs by the US Food and Drug Administration. According to this document, the Food and Drug Administration no longer requires animal testing of a potential drug before clinical trials. As alternatives, computer modeling, organ chips, or organoids can be used^1^. In cancer research, patient-derived organoids (PDOs) are widely used for drug sensitivity screening^2–4^. Gastric cancer (GC) is a common malignancy worldwide, particularly in China^5^. Because of heterogeneity within populations, the efficacy of applied therapies markedly varies. Because PDOs faithfully recapitulate features of the original disease, including its histology, function, and genetic characteristics, they have great potential in pre-clinical studies. In 2009, Sato and colleagues^6^ first established organoids from the small intestine. Since then, tumor organoids derived from stomach cancer^7^, colon cancer^8^, prostate cancer^9^, pancreatic cancer^10^, liver cancer^11^, and breast cancer^12^ have been successfully established and applied in cancer studies.

GC tumor organoids have been used for studying tumor biology^13^, therapeutic drug screening^14^, and drug-resistance mechanisms^15,16^. Gastric organoids from normal mucosa are suitable for the study of mucosal physiology or pathology, mucosal cell functions, and microbial infection^7,17,18^. Organoids are miniaturized and simplified three-dimensional (3D) versions of organs that are self-organized and contain organ-specific cells to exhibit realistic microanatomy; they are also referred to as “living biobanks” that can support medical research through passaging *ex vivo*^19^. However, organoids can undergo only a limited number of cellular passages before losing their cellular vitality. Organoids are constructed in a 3D culture system; therefore, microscopic images of organoids are somewhat different from those acquired through traditional observation methods. The parameters for evaluation of cellular vitality are currently being determined.

With the rapid development of artificial intelligence (AI), new algorithms have been applied to image processing. In the computer vision field, the convolutional neural network (CNN) is a well-known deep learning algorithm. CNNs have been incorporated into medical image processing to assist in disease classification, object detection, and semantic segmentation^20^. In our previous study, efficient tracing of cancer lesions has been achieved through the use of object detection models, which have provided results similar to those obtained through analysis by physicians^21^. Another algorithm based on a visual attention mechanism enables the CNN model to adaptively identify important targets, thus greatly improving target-recognition. Some researchers have combined the attention mechanism with CNN and achieved automatic identification of pulmonary nodules and breast tumors^22^. AI algorithms have provided powerful assistance in medical image studies. For example, Coudray et al.^23^ have used an Inception v3 model to classify subtypes of lung cancer histopathology and to predict 10 commonly mutated genes from pathological images. However, the applications of AI in analyzing organoid images and functional status are limited.

Recently, Bian et al.^24^ built a deep neural network that effectively detects and dynamically tracks organoids throughout the entire culture cycle. However, their research focused on constructing an AI algorithm and did not involve phenotypic analysis. Larsen and coworkers^25^ have established a pan-cancer organoid platform for precision oncology research. They have predicted patient reactions to drugs with neural network based algorithms, and have proposed weak associations of the proliferation marker Ki67 with passaging vitality and biobankability. Abdul and colleagues^26^ have developed an object detection algorithm to distinguish luminal from non-luminal structures in brightfield images of lung epithelial organoids, and have identified the morphological changes that occur after cyclosporine treatment. Okamoto et al.^27^ have used the U-Net semantic segmentation algorithm combined with support vector machine on colorectal cancer organoids, and created an AI-based classifier to group colorectal cancer organoids into 6 subtypes, which are independent of the clinicopathological features of original tumors. The PDO subtypes indicate differences in sensitivity to the RNA polymerase I inhibitor CX-5641.

Because PDOs show a variety of morphological features and have *ex vivo* expansion ability, no criteria for PDO evaluation have been established. Therefore, in this study, we established a system for evaluating PDO vitality according to the staining intensity of senescence-associated β-galactosidase (SA-β-Gal) and the single-cell diameter of a cell suspension, which are reliable parameters for the evaluation of cell senescence^28,29^. We also developed an object detection algorithm that can assist in the evaluation of the expansion ability of organoids.

## Materials and methods

### Organoid construction and passaging

A total of 55 organoids of GC or normal gastric mucosa from 35 patients with GC were constructed. All patients underwent gastrectomy at the Department of General Surgery, Ruijin Hospital of Shanghai Jiaotong University School of Medicine. Tissues were washed twice with PBS buffer containing 1× penicillin/streptomycin (C0222, Beyotime, China) and 100 mg/mL Primocin (A1113802, Thermo Fisher Scientific, USA), and the muscles were removed. The tissues were cut into 1–2 mm^3^ pieces and then digested in 700 μL DMEM buffer containing 1–2 mg/mL collagenase I (40507ES60, Yeasen, China), 0.5–2.5 mg/mL collagenase IV (40510ES60, Yeasen, China), and 0.6–2.4 U/mL dispase II (40104ES60, Yeasen, China) in a water bath with the thermostat set to 37°C, for 1–2 h. Cell suspensions were washed twice with PBS buffer, centrifuged at 1,000 rpm for 5 min, and resuspended with Matrigel (356231, Corning, USA). A 50 μL Matrigel-cell mixture with 5 × 10^4^ cell per well was added dropwise to each well of a pre-warmed 24-well plate. After the drops coagulated, 500 μL of complete organoid medium containing the following essential components was added to each well: advanced DMEM/F12 (12634010, Thermo Fisher Scientific, USA), 1× B27 (17504044, Thermo Fisher Scientific, USA), 1× N2 (17502048, Thermo Fisher Scientific, USA), 10 mM nicotinamide (N0636, Sigma-Aldrich, Germany), 1× L-glutamine (25030081, Thermo Fisher Scientific, USA), 1 mM N-acetyl-L-cysteine (A7250, Sigma-Aldrich, Germany), 200 ng/mL FGF10 (100-26, PeproTech, USA), 50 ng/mL EGF (AF-100-15, PeproTech, USA), 1 nM gastrin (G9145, Sigma-Aldrich, Germany), 2 μM A83-01 (2939, Tocris Bioscience, Britain), 100 ng/mL Wnt3A (5036-WN, R&D Systems, USA), 500 ng/mL R-Spondin1 (120-38, PeproTech, USA), 100 ng/mL Noggin (120-10C, PeproTech, USA), 1 μM prostaglandin E2 (2296, Tocris Bioscience, Britain), and 10 μM Y-27632 (Y0503, Sigma-Aldrich, Germany)^7,30^. The organoids images were captured under an inverted microscope on the 10^th^ day of culture. Normal and GC organoids were passaged at the 14^th^ day of culture. We defined primary cultured cells as P0. Well grown organoids were dissociated into single-cell suspensions by incubation with TrypLE Express (12605010, Thermo Fisher Scientific, USA) at 37°C for 30 min. The cell mixture was washed and embedded in Matrigel at 2,000 cells/μL. After the drops coagulated, 500 μL of complete organoid medium was added to each well. Organoids from both normal mucosa and GC were cultured under the same conditions. This study was approved by the Research Ethics Committee of Shanghai Ruijin Hospital (Approval No. 2019-212). Written informed consent forms were signed by all patients.

### SA-β-Gal assays

A SA-β-Gal staining kit (C0602, Beyotime, China) was used to detect cellular senescence of organoids. At the whole organoid level, organoids at the 14^th^ day were collected into 1.5 mL microcentrifuge tubes without digestion, then fixed with SA-β-Gal fixative solution for 15 min at room temperature. The whole organoids were incubated with SA-β-Gal staining working solution at 37°C overnight. On the next morning, the organoids were washed once with PBS and resuspend in 100 μL PBS. Subsequently, cells were seeded onto glass slides and covered with a coverslip. Under a microscope (100× magnification), images in randomly selected fields were captured. For SA-β-Gal staining at the single-cell level, organoids were digested into single-cell suspensions with TrypLE Express for 30 min. Subsequently, cells were collected into 1.5 mL microcentrifuge tubes and incubated with SA-β-Gal staining working solution at 37°C overnight. On the next morning, organoids were washed once with PBS and resuspend in 100 μL PBS. Subsequently, cells were seeded onto a glass slide and covered with a coverslip. Under a microscope (100× magnification), images were captured in randomly selected fields. The staining intensity of SA-β-Gal was ranked with the following evaluation criteria: 2+, dark green; 1+, light green, and 0+, unstained. Cells with 1+ and 2+ were defined as having SA-β-Gal positivity.

### EdU incorporation assays

Organoids were seeded on 96-well plates for 6 days of culture, then incubated with 5-ethynyl-2’-deoxyuridine (EdU, C0075S, Beyotime, China) for 12 h. Organoids were fixed in 4% paraformaldehyde for 15 min and washed 3 times with 3% BSA. After permeation with immunostaining washing solution for 15 min, organoids were stained with Click Reaction Solution (Click Reaction Buffer 430 μL, CuSO4 20 μL, Azide555 1 μL, and Click Additive Solution 50 μL) for 30 min. Hoechst (C1025, Beyotime, China, 1:1,000) counterstaining was performed for 10 min. Photographs were captured under an inverted fluorescence microscope (TS2R-FL, Nikon, Japan). The nuclei of proliferative cells showed red fluorescence, and non-proliferative cells showed blue fluorescence.

### RNA extraction and PCR analysis

Total RNA was extracted with TRIzol reagent (R401-01-AA, Vazyme, China). RNA (2 mg) was used to synthesize cDNA with HiScript III RT SuperMix (R323-01, Vazyme, China) for qPCR. Quantitative real-time PCR was performed in 10 μL reaction mixtures with ChamQ Universal SYBR qPCR Master Mix (Q711-02, Vazyme, China). RT-PCR was performed in 20 μL reaction mixtures with 10 μL 2× Taq PCR Master Mix, 6 μL ddH_2_O, 1 μL forward primer, 1 μL reverse primer, and 2 μL cDNA (**Table S1**). The products were electrophoresed in 2% agarose gel. The primer sequences were designed and synthesized by Sangon Biotech (Shanghai, China).

### Hematoxylin and eosin (H&E) staining

Whole organoids were collected into 1.5 mL microcentrifuge tubes and centrifuged at 1,500 rpm for 5 min. Organoids were fixed with 4% paraformaldehyde, embedded with 5% agarose and paraffin, and cut into 5 μM thick sections. The sections were dewaxed in xylene for 10 min, absolute ethyl alcohol for 5 min, 95% ethanol 5 min, 85% ethanol for 5 min, 70% ethanol for 5 min, and water for 5 min. Then, sections were stained in hematoxylin 4 min, washed in flowing water for 10 min, differentiated with ethanol hydrochloride for 3 s, washed in flowing water for 10 min, and then eosin stained for 1 min. After staining, the sections were dehydrated again and sealed with neutral resin (G1005, Servicebio, Wuhan, China).

### Immunofluorescence assays

Whole organoids were collected into 1.5 mL microcentrifuge tubes and centrifuged at 1,500 rpm for 5 min. Organoids were fixed with 4% paraformaldehyde, permeabilized with 0.5% Triton X-100 in PBS and incubated in blocking buffer with 5% BSA for 30 min. Subsequently, primary antibodies to p16 (A11651, 1:100 ABclonal, China) and CCNA2 (A19036, 1:100 ABclonal, China), BCL2 (A20777, 1:100, ABclonal, China) were incubated at 4°C overnight. The secondary antibodies (GB22301 and GB22403, 1:200, Servicebio, China) were incubated at room temperature for 50 min, and then counterstaining with DAPI was performed at room temperature for 10 min in the dark. Images were captured with an inverted fluorescence microscope (TS2R-FL, Nikon, Japan).

### Cell cycle assays

Organoids were digested into single cells with TrypLE Express for 30 min, centrifuged at 1,500 rpm for 5 min, washed with precooled PBS, and centrifuged at 1,500 rpm for 5 min. The cells were fixed in 75% ethanol overnight, washed with precooled PBS twice, and centrifuged at 1,500 rpm for 5 min. The cells were resuspended in 300 μL PI/RNase staining solution (550825, BD Biosciences, USA) and incubated in the dark for 30 min, at 37°C. The cell suspension was detected with flow cytometry (BD FACSCalibur, USA).

### Labeling of organoids in images

The organoid images were captured under an inverted microscope (100× magnification) in TIF format. Images with poor quality, low resolution, out of focus organoids or fuzziness were excluded. Image pixels were converted to real size (1 pixel length = 0.833 μm). The images for training model of CBAM-YOLOv3 has been uploaded to the Zenodo database (https://zenodo.org/record/6990360#.YxDJN9NBxPY, DOI 10.5281/zenodo.6988469). The images used for senescence evaluation have been uploaded to the Zenodo database (https://zenodo.org/record/6988469#.YxDJFtNBxPY, DOI 10.5281/zenodo.6990360).

The 2,000 microscopic images captured from 14 samples containing a total of 6,220 organoid spheres were labeled with the LabelImg tool (https://github.com/tzutalin/labelImg). In a classification study of organoids, aging or active organoids were labeled as “active” or “aging” with the LabelImg tool. Images were labeled with priori boxes, then saved in human-readable extensible markup language (XML) format.

### Training the CNN

The study was performed in the following computer environment with hardware comprising an Intel Core i7-10750H CPU, 16G RAM, NVIDIA GeForce RTX 2060, and 6G VRAM. VOC2007 (Visual Object Classes2007) was used for transferring learning images^31,32^, and integrating YOLOv3^33^ and YOLOv4^34^ with visual attention mechanism modules (SENet, CBAM, and ECA). The CBAM-YOLOv3 object detection algorithm was used in the study. the CBAM-YOLOv3 model integrated the CBAM attention mechanism module with the YOLOv3 object detection model, thereby improving feature extraction and object detection ability. Code used in this study has been uploaded to GitHub (https://github.com/ruixinyang08/CBAM-YOLOV3-gastric-organoid, https://github.com/ruixinyang08/CBAM-YOLOV3-organoid-active-and-aging). All computation was run on Google’s Tensorflow1.13.1 and Keras2.2.4 deep learning framework based on Python language^35^. In the training process, the learning rates were 0.001 and 0.0001, and the batch sizes were 2 and 8. Adam was used to optimize the convergence speed and avoid local optima. The MultiboxLoss was used to decrease the imbalance between positive and negative samples. The input sizes of training and evaluation images were 416 × 416 × 3 pixels. The evaluation parameters of the object detection models included mean average precision (mAP), precision, recall, F1, and frames per second^33^. The evaluated parameters were output as organoid average diameter (original measurement value), organoid number, and organoid number × average diameter (No. × Dia.) by the CBAM-YOLOv3 model.

### Statistical analysis

GraphPad Prism 6.0 (GraphPad Prism, RRID: SCR_002798) was used for statistical analysis. Shapiro-Wilk test was used for testing normally distributed data. Student’s t-test (homogeneity of variance) or unpaired t-test with Welch’s correction (heterogeneity of variance) was used for analysis of 2 groups of data. ANOVA or Brown-Forsythe ANOVA was used for data analysis of more than 2 groups. Correlation analysis was performed to compare the SA-β-Gal positivity rate and single-cell diameters, and organoid passages with the AI evaluated results with the CBAM-YOLOv3 algorithm. Each experiment was repeated at least 3 times independently. *P* < 0.05 was considered statistically significant. Data were analyzed statistically with two-sided tests.

## Results

### Phenotypic changes in gastric organoids with passaging

**Figure 1:**
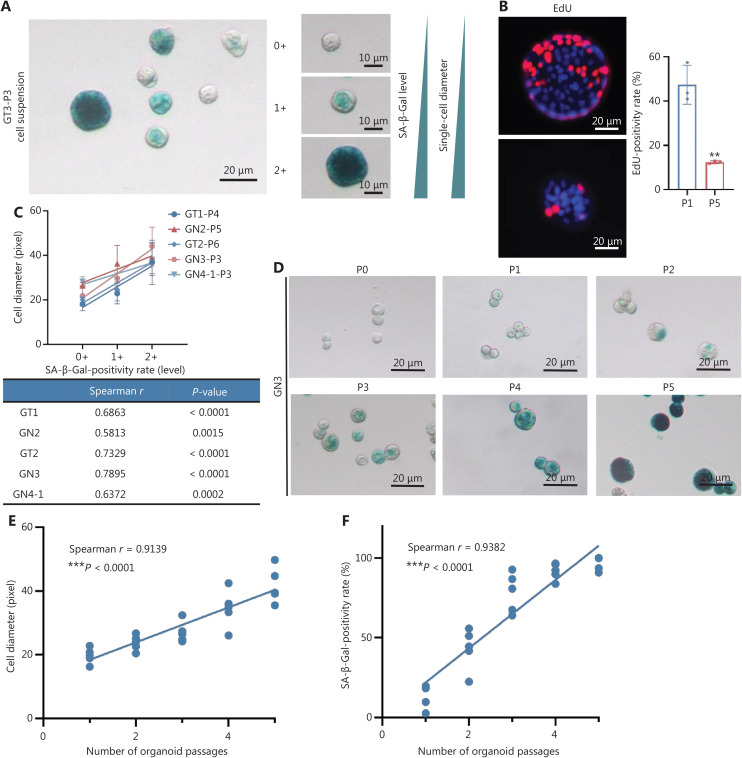
Phenotypic changes in gastric organoids with passaging. (A) The staining intensity of SA-β-Gal is divided into 3 grades: 2+ (dark green), 1+ (light green), and 0+ (unstained). The diameters of single cells gradually increase with the SA-β-Gal staining grade. (B) EdU staining of whole organoids for active (P1) and aging (P5) organoids, ^**^P = 0.0024, *n* = 3, organoid number. (C) Positive correlation between SA-β-Gal staining and single-cell diameter (*n* = 5, case number). (D) Gradually enhanced intensity of SA-β-Gal staining and increased single-cell diameter in passaging from P0 to P5 in GN3. (E) Single-cell diameter positively correlates with extended passaging in all observed gastric mucosa organoids (Spearman *r* = 0.9139, ^***^P < 0.0001). (F) The positivity rate of SA-β-Gal positively correlates with extended passaging in all observed gastric mucosa organoids (Spearman *r* = 0.9382, ^***^P < 0.0001). The experiments were repeated at least 3 times, and results are shown as mean ± *SD*.

In this study, a total of 55 gastric organoids from 35 GC individuals were constructed. The clinicopathological information for these GC patients is presented in **Table 1**. To clarify the phenotypic changes in the organoids with passaging, we measured the single-cell diameter and SA-β-Gal staining intensity of organoids. SA-β-Gal staining positivity was assessed on a scale of 2+ (dark green), 1+ (light green), and 0+ (unstained). Cells with grades of 2+ or 1+ were defined as having SA-β-Gal-positivity (**Figure 1A**). To exclude the influence of cell suspension preparation on cellular activity, we compared the SA-β-Gal staining of cell suspensions with whole organoids without dissociation. Aging organoids exhibited more intense SA-β-Gal staining than active organoids, at both the whole organoid and single-cell levels. In addition, with passaging [to passage (P5)], the organoids exhibited a lower positivity rate of EdU than organoids from an early passage (P1) (**Figure 1B**).

**Table 1:** Clinicopathological information of PDOs of the stomach

Case	*N*	*T*	Gender	Age, years	Location	Borrmann	Histology
1	/	GT1	Female	65	Corpus	I	Adenocarcinoma
2	GN2	GT2	Female	33	Corpus and antrum	IV	Signet-ring cell carcinoma
3	GN3	GT3	Male	65	Antrum	III	Adenocarcinoma
4	GN4-1; 4-2	GT4	Male	72	Cardia	III	Adenocarcinoma
5	GN5	GT5	Female	54	Corpus and antrum	III	Adenocarcinoma
6	GN6	GT6-1; 6-2	Female	68	Antrum	III	Adenocarcinoma
7	GN7	/	Male	71	Corpus and antrum	IV	Adenocarcinoma
8	GN8	GT8	Male	66	Corpus and antrum	IV	Adenocarcinoma
9	GN9	/	Female	62	Corpus and antrum	III	Adenocarcinoma
10	/	GT10	Male	35	Corpus	III	Signet-ring cell carcinoma
11	/	GT11	Male	50	Antrum	I	Adenocarcinoma
12	GN12	/	Male	62	Antrum	III	Adenocarcinoma
13	/	GT13	Male	32	Antrum	III	Signet-ring cell carcinoma
14	/	GT14	Male	74	Cardia	III	Adenocarcinoma
15	GN15	GT15	Female	45	Antrum	III	Signet-ring cell carcinoma
16	/	GT16	Female	49	Corpus and antrum	IV	Adenocarcinoma
17	GN17	GT17	Male	74	Angle	III	Adenocarcinoma
18	/	GT18	Female	49	Corpus	III	Signet-ring cell carcinoma
19	GN19	/	Male	65	Antrum	III	Adenocarcinoma
20	GN20	GT20-1; 20-2	Male	50	Antrum	II	Adenocarcinoma
21	/	GT21	Female	72	Antrum	III	Adenocarcinoma
22	/	GT22	Male	55	Antrum	III	Adenocarcinoma
23	/	GT23	Female	59	Fundus and corpus	IV	Adenocarcinoma
24	/	GT24	Female	50	Corpus	III	Signet-ring cell carcinoma
25	/	GT25	Male	78	Fundus	III	Adenocarcinoma
26	GN26	/	Female	38	Antrum	III	Signet-ring cell carcinoma
27	GN27	/	Male	63	Antrum	III	Adenocarcinoma
28	/	GT28	Female	39	Corpus and antrum	IV	Signet-ring cell carcinoma
29	/	GT29	Male	77	Corpus	III	Adenocarcinoma
30	GN30	GT30	Male	87	Corpus	III	Adenocarcinoma
31	GN31	GT31	Female	78	Corpus	III	Adenocarcinoma
32	GN32-1; 32-2; 32-3	/	Male	61	Antrum	III	Signet-ring cell carcinoma
33	GN33	GT33	Female	63	Corpus	II	Adenocarcinoma
34	GN34-1; 34-2	GT34	Male	68	Cardia	III	Adenocarcinoma
35	GN35	GT35	Male	69	Corpus	III	Adenocarcinoma

We analyzed the relationship between the SA-β-Gal-positivity grade and single-cell diameter in organoids derived from normal mucosa and GC. Higher staining intensity of SA-β-Gal corresponded to larger diameters of single cells (**Figure 1C**). With passaging (P1–P5), the SA-β-Gal-positivity rate and single-cell diameter gradually increased (**Figure 1D**). Moreover, the single-cell diameter gradually increased with passaging in all observed gastric mucosa organoids (Spearman *r* = 0.9139, *P* < 0.0001) (**Figure 1E**). In addition, the increased positivity rate of SA-β-Gal correlated well with extended passaging in all observed gastric mucosa organoids (Spearman *r* = 0.9382, *P* < 0.0001) (**Figure 1F**).

### Evaluation of cellular senescence of organoids with the CBAM-YOLOv3 algorithm

**Figure 2:**
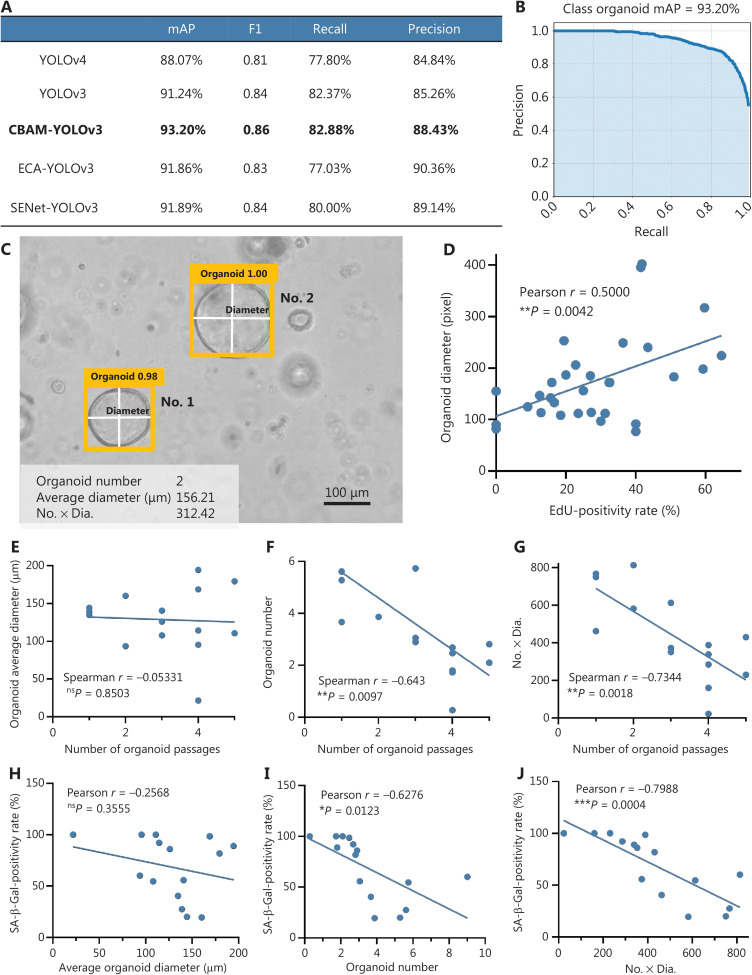
Evaluation of cellular senescence of organoids with the CBAM-YOLOv3 algorithm. (A) Comparison of the training effects of several AI models, revealing optimal performance for CBAM-YOLOv3. (B) The mAP of the CBAM-YOLOv3 model reaches 93.20% in the test set. (C) Output results of organoid evaluation by CBAM-YOLOv3, including the 3 parameters of organoid average diameter, organoid number, and No. × Dia. (D) The proportion of EdU incorporated into organoids positively correlates with the average diameter of organoids (Pearson *r* = 0.5000, ^**^P = 0.0042, *n* = 31, number of organoids). (E) The organoid average diameter from P1 to P5 is not significantly correlated with extended passaging (Spearman *r* = −0.05331, ^ns^*P* = 0.8503). (F) The organoid average number is positively correlated with extended passaging (Spearman *r* = −0.6430, ^**^P = 0.0097). (G) No. × Dia. of organoids is positively correlated with extended passaging (Spearman *r* = −0.7344, ^**^P = 0.0018). (H) The organoid average diameter is not significantly correlated with SA-β-Gal positivity (Pearson *r* = −0.2568, ^ns^*P* = 0.3555). (I) The organoid average number is positively correlated with the SA-β-Gal positivity rate (Pearson *r* = −0.6276, ^*^P = 0.0123). (J) No. × Dia. of organoids is positively correlated with the SA-β-Gal positivity rate (Spearman *r* = −0.7988, ^***^P = 0.0004). Case number: *n* = 15. The experiments were repeated at least 3 times, and results are shown as mean ± *SD*.

We used microscopic images captured from organoids for the AI study. The 2,000 images were divided into a training set and test set (1,600:400) in a 4:1 ratio. Each organoid in the images was manually labeled with the LabelImg tool. A total of 6,220 organoid spheres were marked (training set:test set, 5,040:1,180). We compared the object detection algorithms of YOLOv3 and YOLOv4, and found that the parameters of mAP, F1, recall, and precision in YOLOv3 (91.24%, 0.84, 82.37%, and 85.26%, respectively) were better than those of YOLOv4 (88.07%, 0.81, 77.80%, and 84.84%, respectively) (**Figure 2A**). Therefore, we integrated the convolutional block attention module (CBAM) in the YOLOv3 object detection algorithm to improve feature extraction (**Supplementary Figure S1**). The parameters of mAP, F1, recall, and precision in CBAM-YOLOv3 were clearly optimized (93.20%, 0.86, 82.88%, and 88.43%, respectively) (**Figure 2A and 2B**). The number of frames per second for the CBAM-YOLOv3 model was 19.65, and was markedly higher than that with manual analysis (0.077). The CBAM-YOLOv3 model accurately extracted the organoid average diameter, organoid number, and No. × Dia., and output these parameters for images (**Figure 2C**). To analyze cell proliferation activity, we performed EdU incorporation assays in culture and found that the average diameter of organoids moderately correlated with cell proliferation activity, on the basis of an increased EdU incorporation rate (**Figure 2D**, Pearson *r* = 0.5000, *P* = 0.0042).

In the CBAM-YOLOv3 algorithm evaluation, the average diameter of organoids did not substantial change as the number passages was extended from P1 to P5 (Spearman *r* = −0.05331, *P* = 0.8503, **Figure 2E**), whereas the organoid number was significantly correlated with extended passaging (Spearman *r* = −0.6430, *P* = 0.0097, **Figure 2F**). Moreover, No. × Dia. clearly decreased with passaging (Spearman *r* = −0.7344, *P* = 0.0018; **Figure 2G**). We examined the correlations of these parameters with SA-β-Gal staining and found that the organoid average diameter did not significantly correlate with the SA-β-Gal positivity rate (Pearson *r* = −0.2568, *P* = 0.3555; **Figure 2H**), whereas organoid number (Pearson *r* = −0.6276, *P* = 0.0123; **Figure 2I**) and No. × Dia. (Pearson *r* = −0.7988, *P* = 0.0004; **Figure 2J**) significantly correlated with the SA-β-Gal positivity rate.

### Limited passaging of organoids derived from normal mucosa

**Figure 3:**
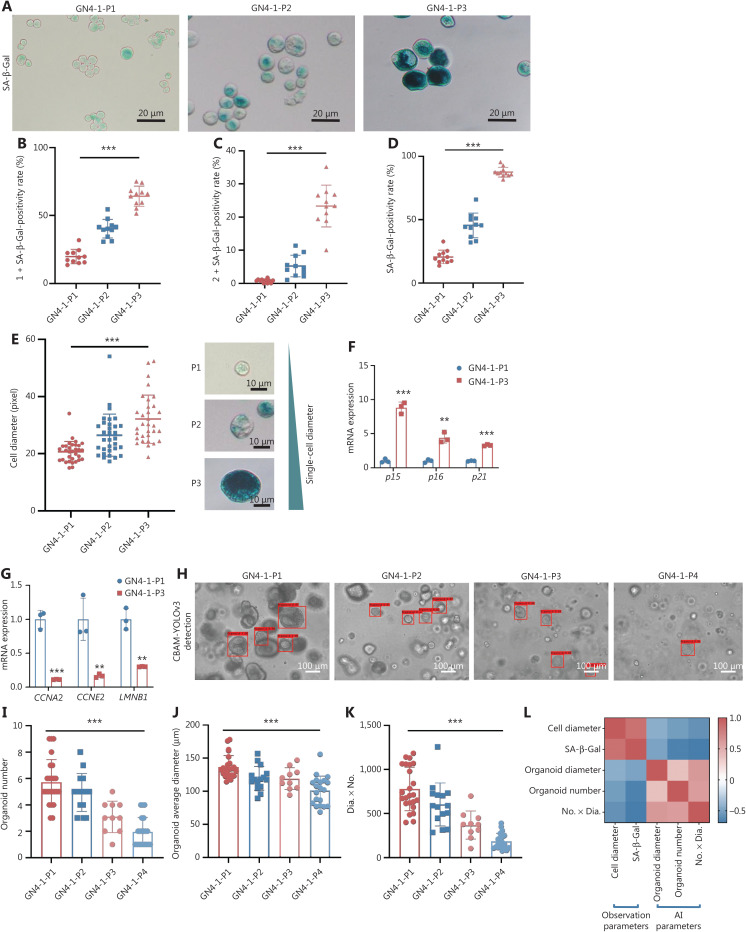
Limited passaging of organoids derived from normal mucosa. (A) In the GN4 organoid, the staining intensity of SA-β-Gal in cell suspension gradually increases from P1 to P3. (B–D) Gradually increased SA-β-Gal-positivity rates of 1+ (^***^P < 0.0001), 2+ (^***^P < 0.0001), and total (^***^P < 0.0001) from P1 to P3. (E) Increased single-cell diameter from P1 to P3 (^***^P < 0.0001); *n* = 3, random field. (F) Increased mRNA levels of the cell cycle inhibitors *p15* (^***^P = 0.0001), *p16* (^**^P = 0.0018), and *p21* (^***^P < 0.0001). (G) Decreased mRNA levels of the cell cycle enhancers *CCNA2* (^***^P = 0.0003), *CCNE2* (^**^P = 0.0099), and *LMNB1* (^**^P = 0.0016) from P1 to P3. (H) Evaluative outputs of the CBAM-YOLOv3 model, presented from P1 to P4. (I) Evaluative outputs of organoid numbers from P1 to P4, obtained from the CBAM-YOLOv3 model (^***^P < 0.0001). (J) Evaluative outputs of organoid average diameter from P1 to P4, obtained from the CBAM-YOLOv3 model (^***^P < 0.0001). (K) Evaluative outputs of organoid No. × Dia. from P1 to P4, obtained from the CBAM-YOLOv3 model (^***^P < 0.0001). (L) Correlation analysis of observation parameters of SA-β-Gal and single-cell diameter with evaluative parameters from the CBAM-YOLOv3 algorithm. The red box indicates positive correlation, and the blue box indicates negative correlation. The stronger the coefficients, the darker the color; *n* = 4, passage number. The experiments were repeated at least 3 times, and results are shown as mean ± *SD*.

To clarify the features of passaged organoids derived from normal mucosa, we examined the phenotypic changes in 10 cases of organoids from normal mucosae (**Table S2**). Cellular senescence was observed in 6 of 10 cases (60%) at P5. For example, in the case of GN4, the staining intensity of SA-β-Gal gradually increased from P1 to P3 (**Figure 3A**). The positivity rate of 1+SA-β-Gal (**Figure 3B**), 2+SA-β-Gal (**Figure 3C**), and total SA-β-Gal (**Figure 3D**) gradually increased. The single-cell diameters of cell suspensions also gradually increased (**Figure 3E**). The gene expression levels of cell cycle inhibitors including cyclin dependent kinase inhibitor 2B (*p15*), cyclin dependent kinase inhibitor 2A (*p16*), and cyclin dependent kinase inhibitor 1A (*p21*) increased (**Figure 3F**), whereas that of cell cycle enhancers such as cyclin A2 (*CCNA2*), cyclin E2 (*CCNE2*), and lamin B1 (*LMNB1*) decreased from P0 to P3 (**Figure 3G**). Similar changes were observed in other cases of organoids from normal mucosae (**Table S2, Supplementary Figures S2–S4**). In addition, the protein expression level of p16 increased, whereas that of CCNA2 decreased, from P0 to P3. To explore the effect of apoptosis on cell senescence, we examined the protein expression level of BCL2, an anti-apoptotic factor, and observed increased expression from P0 to P3 (**Supplementary Figure S5**).

We evaluated the organoid number, organoid average diameter, and No. × Dia. of P1 to P4 for the GN4 case with the CBAM-YOLOv3 algorithm (**Figure 3H**). Gradual decreases in organoid number (**Figure 3I**), organoid average diameter (**Figure 3J**), and No. × Dia. (**Figure 3K**) were observed. In addition, the phenotypic parameters of the SA-β-Gal positivity rate and single-cell diameter correlated well with the AI evaluative parameters of organoid number, organoid average diameter, and No.×Dia. (**Figure 3L**). The positivity rate of SA-β-Gal was positively associated with single-cell diameter (Pearson *r* = 0.8560, *P* < 0.0001), but negatively associated with organoid average diameter (Pearson *r* = −0.4925, *P* = 0.0060), organoid number (Pearson *r* = −0.6687, *P* < 0.0001), and No. × Dia. (Pearson *r* = −0.6923, *P* < 0.0001). The single-cell diameter was also negatively associated with organoid average diameter (Pearson *r* = −0.4344, *P* = 0.0164), organoid number (Pearson *r* = −0.5156, *P* = 0.0035), and No. × Dia. (Pearson *r* = −0.5821, *P* = 0.0007). Similar changes were observed in other cases (**Supplementary Figures S2–S4**).

### Potentially unlimited passaging of organoids derived from GCs

**Figure 4:**
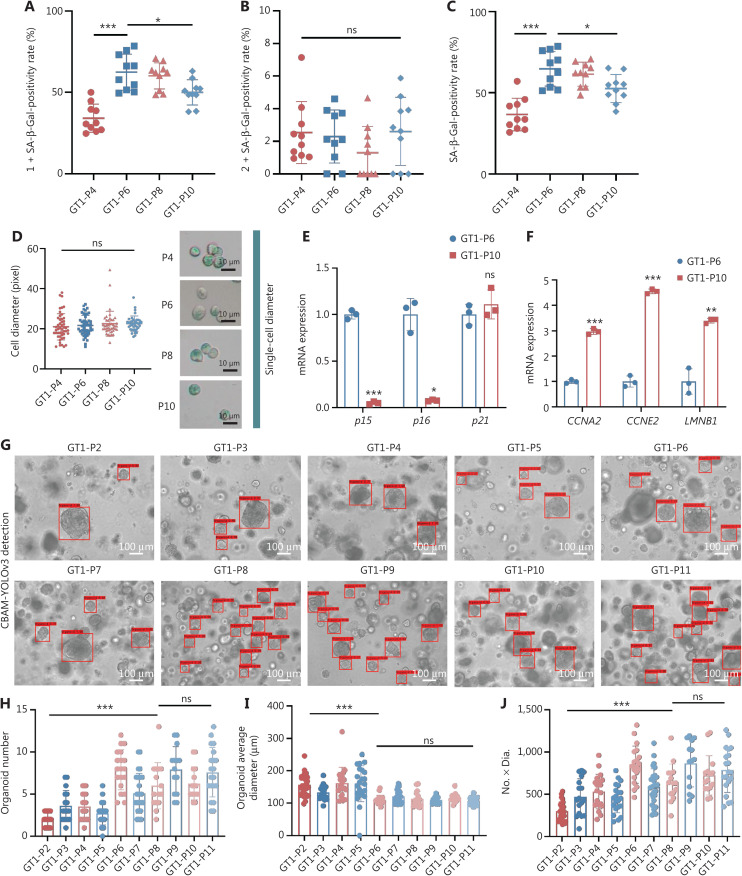
Potential unlimited passaging of organoids derived from gastric cancer. (A) The SA-β-Gal-positivity rate of 1+ grade increases from P4 to P6 (^***^P < 0.0001), then slightly decreases from P8 to P10 (^*^P = 0.0113). (B) The SA-β-Gal-positivity rate of 2+ grade remains low (^ns^*P* = 0.3611). (C) The positivity rate of total SA-β-Gal slightly increases from P4 to P6 (^*^P = 0.0156), then decreases from P8 to P10 (^***^P < 0.0001). (D) The single-cell diameter of GT1 organoids from P4 to P10 remains stable (^ns^*P* = 0.5125); *n* = 4, random field. (E) The mRNA levels of *p15* and *p16* decrease, but that of *p21* does not change significantly (^***^P < 0.0001, ^*^P = 0.0114, and ^ns^*P* = 0.3783). (F) The mRNA levels of *CCNA2*, *CCNE2*, and *LMNB1* increase from P6 to P10 (^***^P < 0.0001, ^***^P < 0.0001, and ^**^P = 0.0012); *n* = 2, passage number. (G) Evaluative outputs of the CBAM-YOLOv3 model from P2 to P11. (H) With passaging from P2 to P8, the organoid numbers fluctuate (^***^P < 0.0001), then remain stable from P9 to P11 (^ns^*P* = 0.1204). (I) The organoid average diameter fluctuates from P2 to P5 (^***^P < 0.0001), then remains stable from P6 to P11 (^ns^*P* = 0.1624). (J) The parameter of No. × Dia. shows a trend similar to that of organoid number (^***^P < 0.0001, ^ns^*P* = 0.1067); *n* = 10, passage number. The experiments were repeated at least 3 times and are shown as mean ± *SD*.

Ten cases of organoids derived from GCs revealed heterogeneous cellular vitality. Four of the 10 cases (40%) showed senescence characteristics to some extent, and passaging ended at P5 or P6, whereas the other 6 cases (60%) showed good long-term passaging from passages 25 to passages 45 (with culturing for as many as 511 days) (**Table S2**). For example, in the case of GT1, the SA-β-Gal staining intensity slightly increased from P4 to P6, then decreased from P6 to P10. The SA-β-Gal-positivity rate of 1+ grade slightly increased from P4 to P6, then decreased after P6 (**Figure 4A**). The SA-β-Gal-positivity rate of 2+ grade remained stable at a low level (**Figure 4B**). The total SA-β-Gal-positivity rate (**Figure 4C**) increased from P4 to P6, then decreased after P6. The single-cell diameters of organoids remained stable from P4 to P10 (**Figure 4D**). The expression levels of *p15* and *p16* decreased (**Figure 4E**), whereas those of *CCNA2*, *CCNE2*, and *LMNB1* increased, from P6 to P10 (**Figure 4F**). On the basis of CBAM-YOLOv3 evaluation, the AI model successfully output the organoid parameters from P2 to P11 (**Figure 4G**). With passaging, the organoid number (**Figure 4H**) slowly fluctuated, then increased (P2 to P8), and remained stable at P8 to P11. The organoid average diameter (**Figure 4I**) fluctuated (from P2 to P6), then remained stable at P6 to P11. The No. × Dia. (**Figure 4J**) exhibited trends similar to those of organoid number.

**Figure 5:**
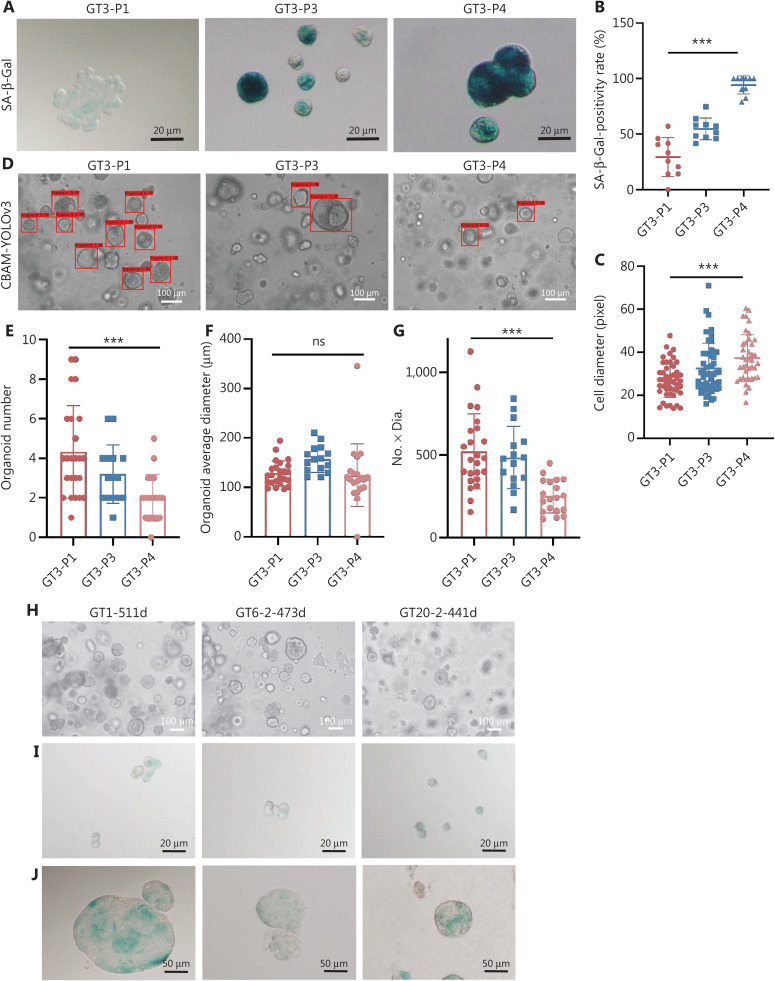
Heterogeneous features of organoids derived from gastric cancer. (A) The staining intensity of SA-β-Gal in case GT3 clearly increases with extended passaging from P1 to P4. (B) The total positivity rate of SA-β-Gal staining increases from P1 to P4, and reaches nearly 100% at P4 (^***^P < 0.0001). (C) The single-cell diameter increases from P1 to P4 (^***^P < 0.0001). (D) Evaluative outputs of the CBAM-YOLOv3 model for GT3 organoids from P1 to P4. (E) Decreased organoid number with increased passaging from P1 to P4 (^***^P < 0.0001). (F) The evaluative outputs for organoid average diameter are relatively stable (^ns^*P* = 0.0745). (G) Decreased evaluative outputs for No. × Dia. (^***^P < 0.0001). (H) Three cases of cancer organoids are passaged for a long time and retain active proliferation morphology (GT1 for 511 days, GT6-2 for 473 days, and GT20-2 for 441 days). (I) Single cell suspensions from long-term cultured organoids show weakened SA-β-Gal staining. (J) Whole organoids derived from long-term cultured organoids show weakened SA-β-Gal staining. *n* = 3, passage number. The experiments were repeated at least 3 times, and results are shown as mean ± *SD*.

In contrast, the senescence features of GT3 were heterogeneous with respect to those of GT1. The staining intensity of SA-β-Gal clearly increased from P1 to P4 (**Figure 5A**), and the total positivity rate of SA-β-Gal staining reached nearly 100% at P4 (**Figure 5B**), thus resulting in difficult passaging in this condition. The single-cell diameter of GT3 also increased from P1 to P4 (**Figure 5C**). The evaluative outputs of the CBAM-YOLOv3 model for GT3 organoids from P1 to P4 are presented in **Figure 5D**. With extension of passaging from P1 to P4, the organoid number decreased (**Figure 5E**), the organoid average diameter was relatively stable (**Figure 5F**), and the No. × Dia. decreased (**Figure 5G**). According to correlation analysis, the SA-β-Gal staining and single-cell diameter showed satisfactory correlations with the evaluative parameters of the CBAM-YOLOv3 algorithm for GT3 organoids. With increasing passaging, the staining intensity of SA-β-Gal positively correlated with single-cell diameter. The positivity rate of SA-β-Gal did not correlate with the organoid average diameter output according to the CBAM-YOLOv3 model, but significantly negatively correlated with the parameters of organoid number and No. × Dia. The single-cell diameter did not correlate with the organoid average diameter, but negatively correlated with the parameters of organoid number and No. × Dia. The phenotypic changes in the GT4 organoids showed trends similar to those of the GT3 organoids (**Supplementary Figure S6**).

To characterize the phenotypic changes in long-term cultured organoids derived from GCs, we serially passaged 3 cases of cancer organoids in our laboratory (GT1 for 511 days, GT6-2 for 473 days, and GT20-2 for 441 days). The organoids maintained active proliferation morphology (**Figure 5H**) and showed weakened SA-β-Gal staining at both the single cell suspension level (**Figure 5I**) and the whole organoid level (**Figure 5J**).

### Classification of active or aging organoids with the CBAM-YOLOv3 algorithm

**Figure 6:**
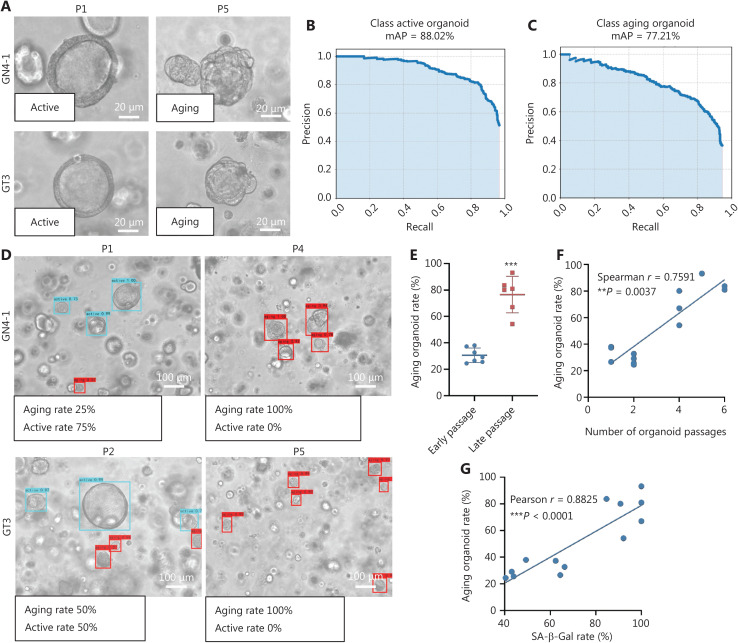
Classification of active or aging organoids with the CBAM-YOLOv3 algorithm. (A) Organoids in early stages of P1 exhibit regular structures, smooth boundaries, and thick-walled single lumen glands, whereas organoids in P5 show a disorganized structure, rough borders, and a loss of glandular structures. (B) The CBAM-YOLOv3 model accurately evaluates active organoids, with an mAP of 88.02%. (C) The CBAM-YOLOv3 model evaluates senescent organoids, with an mAP of 77.21%. (D) The CBAM-YOLOv3 model can output the proportions of active organoids and aging organoids. (E) CBAM-YOLOv3 model evaluation of the aging organoid rate in early passages (P1–P2) and late passages (P4–P5) in the prospective validation dataset, ^***^P < 0.0001. (F) The proportion of aging organoids is positively associated with extended passaging (Spearman *r* = 0.7591, ^**^P = 0.0037). (G) The proportion of aging organoids is positively associated with the positivity rate of SA-β-Gal staining (Pearson *r* = 0.8825, ^***^P < 0.0001); *n* = 13, case number. The experiments were repeated at least 3 times, and results are shown as mean ± *SD*.

Under microscopic examination, the early-stage organoids in P1 to P2 exhibited regular structures, smooth boundaries, and thick-walled single lumen glands, in contrast to organoids at P4 or later (**Figure 6A**). The senescent organoids exhibited a disorganized structure, rough borders, loss of glandular structures, and small glands. Compared with early passage organoids (P1), the senescent organoids (P4) were arrested in G0/G1 phase, according to flow cytometric analysis (**Supplementary Figure S7**). We used images from P1 to P2 to indicate proliferative organoids (active), and images from P4 or later to indicate senescent organoids (aging). We captured 900 microscopic images (at the same magnification) on the 10^th^ day for all included cases in a 4:1 training set:test set ratio. The CBAM-YOLOv3 algorithm accurately evaluated active organoids, with an mAP of 88.02% (**Figure 6B**), and aging organoids, with an mAP of 77.21% (**Figure 6C**). The overall mAP of the CBAM-YOLOv3 algorithm was 82.62%. The AI program also output the proportion of active organoids and aging organoids (**Figure 6D**). We prospectively validated organoids from 13 separate cases with the CBAM-YOLOv3 algorithm. Aging organoids increased with passaging from early stages (P1 to P2) to late stages (P4 to P5) (**Figure 6E**). The proportion of aging organoids was positively associated with extended passaging (Spearman *r* = 0.7591, *P* = 0.0037) (**Figure 6F**) and the positivity rate of SA-β-Gal staining (Pearson *r* = 0.8825, *P* = 0.0001) (**Figure 6G**).

## Discussion

No available evidence has indicated how many times organoids can be passaged without loss of basic biological functions. The present study systematically investigated the relationship between organoid passaging and cellular senescence for normal gastric epithelium and GCs. We analyzed the cellular senescence parameters of SA-β-Gal staining and single-cell diameter, which accurately reflect the senescence phenotype with extended passaging^28^. In addition, we examined mRNA levels of the cell cycle inhibitors *p15*, *p16*, and *p21*, as well as the cell cycle enhancers *CCNA2*, *CCNE2*, and *LMNB1*, which together have been proposed as genetic parameters in aging studies^29^. Given that the evaluation of the above parameters is laborious and time-consuming, we performed AI-assisted evaluation to determine the cellular senescence of organoids. We captured large numbers of organoid images and trained a computer program. A CBAM-YOLOv3 object detection model was constructed and used to evaluate cellular senescence. As compared with traditional phenotypic indicators, the CBAM-YOLOv3 model efficiently evaluated active organoids and aging organoids, and yielded results compatible to those from SA-β-Gal staining and single-cell diameter measurement. In general, when the aging organoid rate exceeds 80% in CBAM-YOLOv3 evaluation, the organoid sample should be considered poor quality and may be difficult to use in subsequent experiments. Our study provides evidence that an AI algorithm aids in gauging the growth status of gastric organoids, and can greatly decrease labor cost and time.

AI algorithms have been widely used in the processing of medical images, such as CT images, ultrasound images, endoscopic images, and pathological images^36–40^. However, limited studies have used AI algorithms for organoids. Bian and colleagues^24^ have constructed an AI model to track organoids with a 3D culture system. Abdul and colleagues^26^ have used deep learning techniques to classify polarized and non-polarized lung epithelial organoids in microscopic images. Currently, no research has used AI algorithms for the evaluation of organoid vitality. The CBAM-YOLOv3 model had optimal performance, with an mAP of 93.20% for organoid recognition. The CBAM-YOLOv3 model accurately evaluated the organoid average diameter, organoid number, and No. × Dia. parameters for evaluating the senescent state of organoids. The parameter No. × Dia. showed optimal performance in evaluation of cell senescence in organoids.

Our research provides a valuable method for evaluating the translational use of organoid experimental models. We observed the aging tendencies of organoids derived from normal mucosa with passaging. We recommend that all experiments in normal gastric organoids be conducted before P5, except in aging-associated studies. Organoids derived from GC showed heterogeneity in cellular activity: some tumor organoids were sustainably passaged without clear senescent phenotypes (with culturing for as many as 511 days), whereas several cases showed senescent tendencies similar to those of organoids from normal mucosa. We speculated that this heterogeneity might be associated with lower tumor purity in cancer organoids, because some normal epithelial organoids might overgrow and affect the growth of tumor organoids^41,42^. In contrast, if the organoids derived from cancers were to have high purity of cancer cells, the organoids would have potential for unlimited passaging *ex vivo*, thus supporting the cancer hallmarks proposed by Hanahan^43^. Cells of cancer organoids may acquire capabilities to sustain proliferative signaling, evade growth suppressors, resist cell death, and enable replicative immortality. Recently, this infinite proliferation potential has been proposed to support the utility of PDOs in establishing cell lines to serve as experimental models^44,45^.

Currently, systematic research on whether the repeated passaging of organoids influences cellular vitality is lacking. The current findings should help researchers obtain reliable experimental data for considering the use of organoids as experimental tool. Because the AI algorithm can evaluate the degree aging of organoids on the basis of only brightfield images captured in organoid culture, the time required for preparing single-cell suspensions and performing SA-β-Gal staining is greatly decreased, and the working procedures are improved.

The current study has several limitations. First, owing to the high heterogeneity of GCs, using a unified standard to evaluate the cellular senescent state is difficult. We will include a similarity analysis algorithm for comparing organoids at different passages in a future study. Second, the images collected in our study were static images. We hope to integrate the AI program into microscopes’ image capture procedures to achieve real-time evaluation. Third, this study focused on evaluating the cellular senescence of only gastric organoids. However, we plan to extend this model to evaluate the cellular senescence of other types of organoids.

## Conclusions

Researchers should be aware that the cellular vitality of organoids changes with passaging. The CBAM-YOLOv3 algorithm is useful for evaluating the cellular senescence status for gastric organoids. Experimental studies using organoid models of normal gastric mucosa should be conducted within 5 passages. Tumor organoids demonstrated potential for unlimited proliferation and passaging. Because the AI algorithm can evaluate the degree of aging of organoids by using only brightfield images, the time required for preparing single-cell suspensions and SA-β-Gal staining is greatly decreased, thereby diminishing the time required for quality control, and improving working procedures for living biobank administration.

## Data Availability

The images used for training the CBAM-YOLOv3 model have been uploaded to the Zenodo database (https://zenodo.org/record/6990360#.YxDJN9NBxPY, DOI 10.5281/zenodo.6988469). The images for senescence evaluation have been uploaded to the Zenodo database (https://zenodo.org/record/6988469#.YxDJFtNBxPY, DOI 10.5281/zenodo.6990360). Code used in this study has been uploaded to GitHub (https://github.com/ruixinyang08/CBAM-YOLOV3-gastric-organoid, https://github.com/ruixinyang08/CBAM-YOLOV3-organoid-active-and-aging).
