# Supplementary material for: A quick and reliable image-based AI algorithm for evaluating cellular senescence of gastric organoids
Source: Cancer Biol Med. 2023 Jun 30;20(7):519–36. doi: 10.20892/j.issn.2095-3941.2023.0099 (PMC10466441; doi:10.20892/j.issn.2095-3941.2023.0099)
Supplement: Supplementary file 1 [file cbm-20-519-s001.pdf]

Supplementary materials

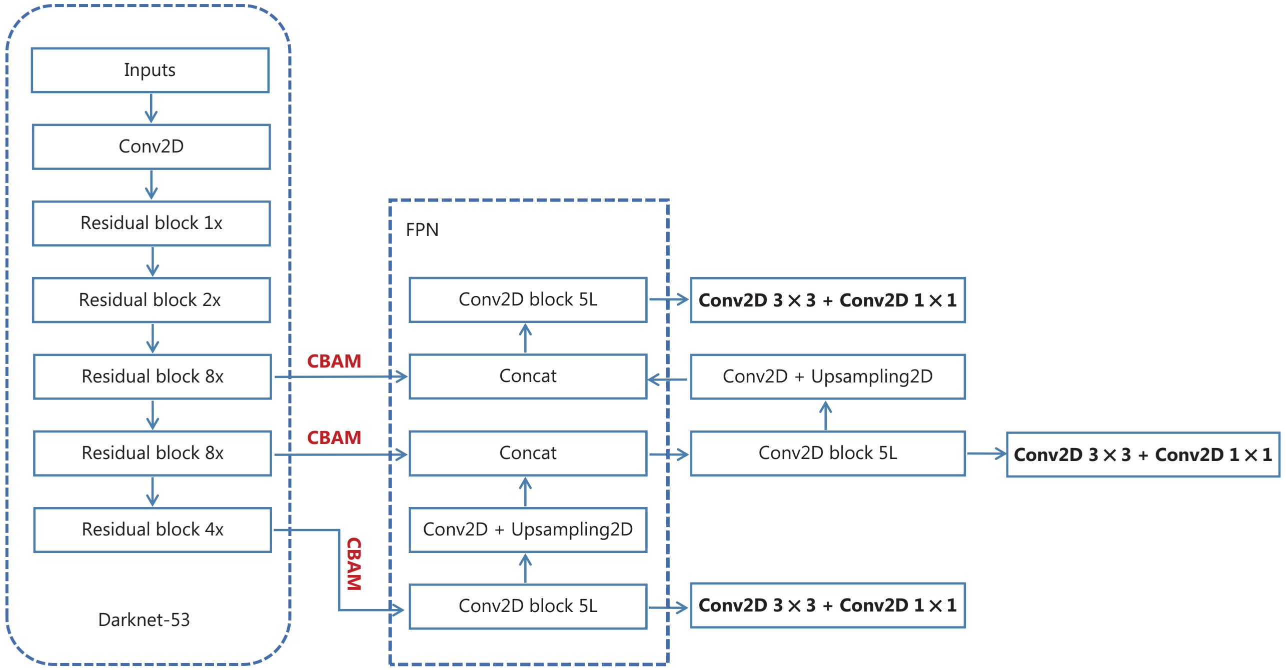

Figure S1 Integration of the CBAM attention mechanism module with the YOLOv3 object detection model. The backbone feature extraction network is Darknet-53 in the YOLOv3 model. The attention mechanism module is added in front of the feature pyramid networks (FPN).

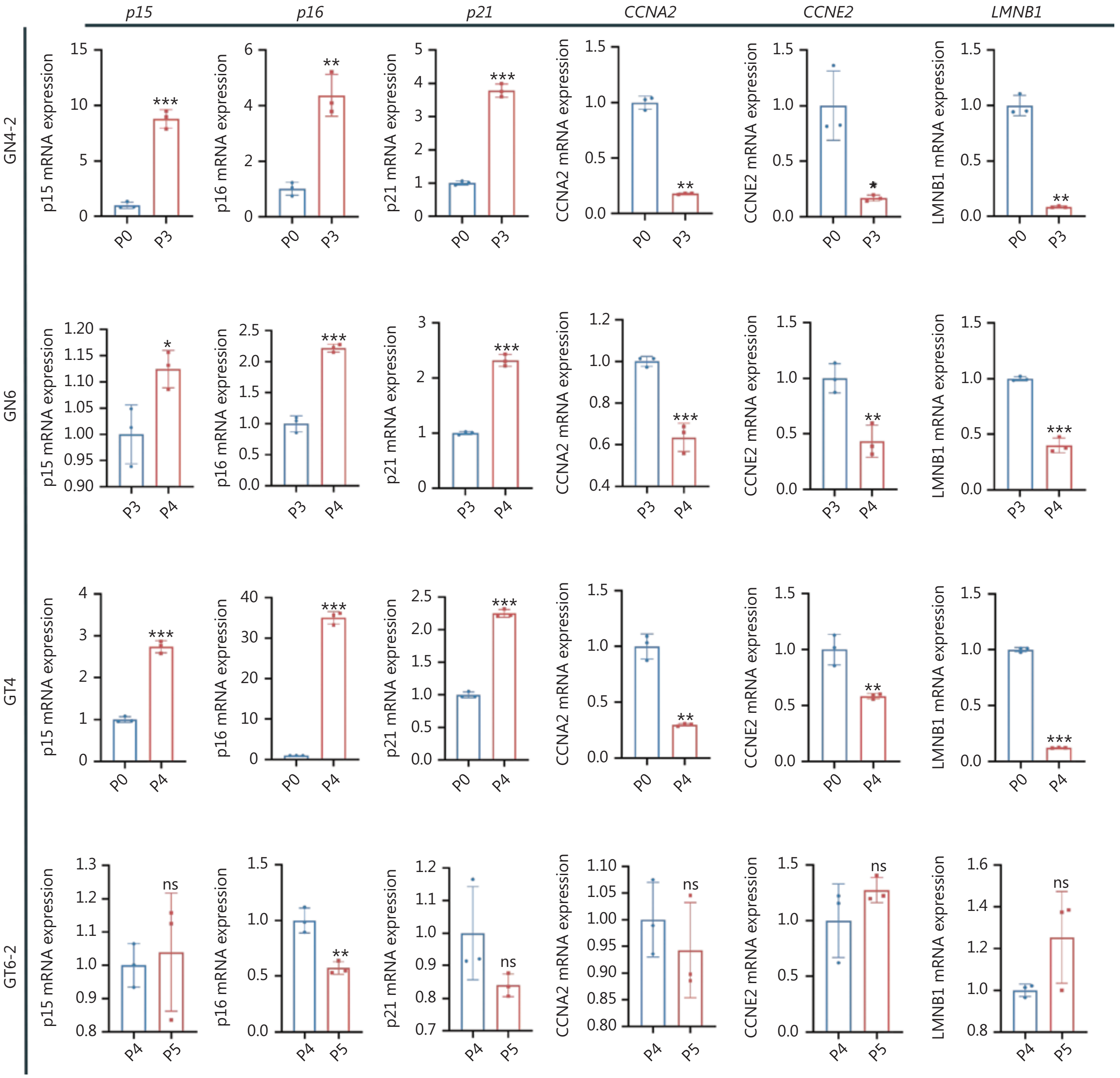

Figure S2 The mRNA expression levels of cell cycle inhibitors and cell cycle enhancers of organoids from GN4-2, GN6, GT4, and GT6-2 in different passages. The cases of GN4-2, GN6, and GT4 reveal similar trends in the mRNA expression of *p15*, *p16*, *p21*, *CCNA2*, *CCNE2*, and *LMNB1* with passaging. The mRNA expression levels of *p15*, *p16*, and *p21* are up-regulated, whereas those of *CCNA2*, *CCNE2*, and *LMNB1* are down-regulated (GN4-2 ***P = 0.0001, **P = 0.0018, ***P < 0.0001, **P = 0.0016, *P = 0.0427, **P = 0.0032; GN6 *P = 0.0317, ***P = 0.0001, ***P < 0.0001, ***P = 0.0009, **P = 0.0075, ***P = 0.0001; GT4 ***P < 0.0001, ***P = 0.0007, ***P < 0.0001, **P = 0.0080, **P = 0.0066, ***P = 0.0001). In GT6-2, the mRNA expression levels of *p21*, *p15*, *CCNA2*, *CCNE2*, and *LMNB1* do not significantly differ, except for *p16*, which is slightly down-regulated (^{ns}P = 0.7337, **P = 0.0041, ^{ns}P = 0.1329, ^{ns}P = 0.4325, ^{ns}P = 0.2443, ^{ns}P = 0.1818). The experiments were repeated 3 times, and results are shown as mean ± *SD*.

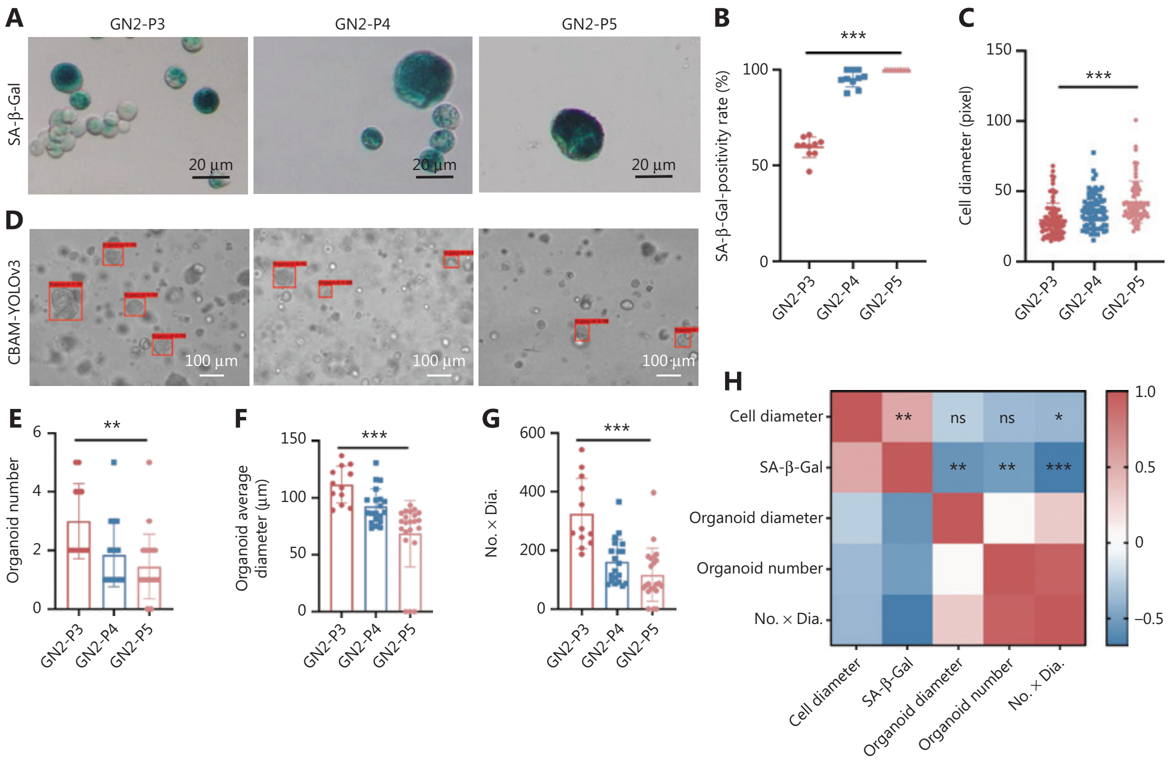

Figure S3 Senescence analysis of organoids from GN2. (A) Increased staining intensity of SA-β-Gal with extended passaging from P3 to P5. (B) Increased total positivity rate of SA-β-Gal from P3 to P5 (***P < 0.0001). (C) Increased single-cell diameter from P3 to P5 (**P = 0.0005). (D) Evaluative outputs of the CBAM-YOLOv3 model from P3 to P5. (E) Evaluative results of organoid number, output by the CBAM-YOLOv3 model. Organoid numbers gradually decrease with extended passaging from P3 to P5 (***P = 0.0017). (F) Decreased organoid average diameter (***P < 0.0001). (G) Decreased evaluative results of No. × Dia. with extended passaging from P3 to P5 (***P < 0.0001). (H) Correlation analysis of observation parameters of SA-β-Gal staining and single-cell diameter with evaluative parameters by using the CBAM-YOLOv3 algorithm. The red box represents positive correlation, and the blue box represents negative correlation. The stronger the coefficients, the darker the color. The single-cell diameter positively correlates with the SA-β-Gal-positivity rate (Pearson r = 0.5262, **P = 0.0028). The SA-β-Gal-positivity rate negatively correlates with organoid average diameter (Pearson r = −0.5595, **P = 0.0013), organoid number (Pearson r = −0.5123, **P = 0.0038), and No. × Dia. (Pearson r = −0.6786, ***P < 0.0001). No correlation is observed between single-cell diameter and organoid average diameter (Pearson r = −0.2538, ^{ns}P = 0.1760) or organoid number (Pearson r = −0.3585, ^{ns}P = 0.0517). Single-cell diameter negatively correlates with No. × Dia. (Pearson r = −0.3786, *P = 0.0391). n = 3, passage number. The experiments were repeated 3 times, and results are shown as mean ± SD.

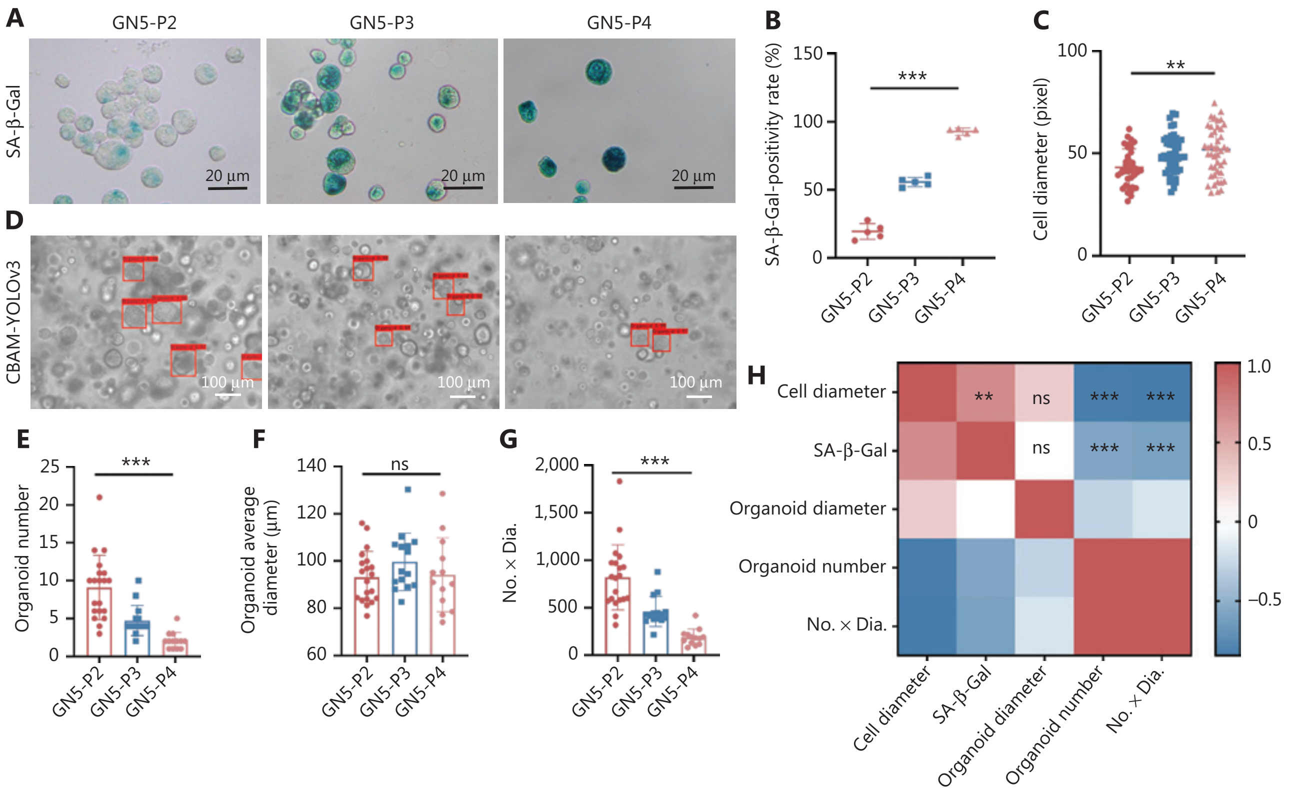

Figure S4 Senescence analysis of organoids from GN5. (A) Increased staining intensity of SA-β-Gal with extended passaging from P2 to P4. (B) Increased total positivity rate of SA-β-Gal from P2 to P4 (***P < 0.0001). (C) Increased single-cell diameter from P2 to P4 (**P = 0.0041). (D) Evaluative outputs using the CBAM-YOLOv3 model from P2 to P4. (E) Decreased organoid number with extended passaging from P2 to P4 (***P < 0.0001). (F) The organoid average diameter remains stable (^{ns}P = 0.3104). (G) Decreased No. × Dia. from P2 to P4 (***P < 0.0001). (H) Correlation analysis of observation parameters of SA-β-Gal staining and single-cell diameter with evaluative parameters by using the CBAM-YOLOv3 algorithm. The red box represents positive correlation, and the blue box represents negative correlation. The stronger the coefficients, the darker the color. The single-cell diameter positively correlates with SA-β-Gal-positivity rate (Pearson r = 0.6918, **P = 0.0043). No correlation is observed between the SA-β-Gal-positivity rate and organoid average diameter (Pearson r = −0.0084, ^{ns}P = 0.9648). The SA-β-Gal-positivity rate negatively correlates with organoid number (Pearson r = −0.5711, ***P = 0.0010) and No. × Dia. (Pearson r = −0.5970, ***P < 0.0001). The single-cell diameter is not correlated with organoid average diameter (Pearson r = 0.3190, ^{ns}P = 0.2464), but is negatively correlated with organoid number (Pearson r = −0.8408, ***P < 0.0001) and No. × Dia. (Pearson r = −0.8437, ***P < 0.0001). n = 3, passage number. The experiments were repeated 3 times, and results are shown as mean ± SD.

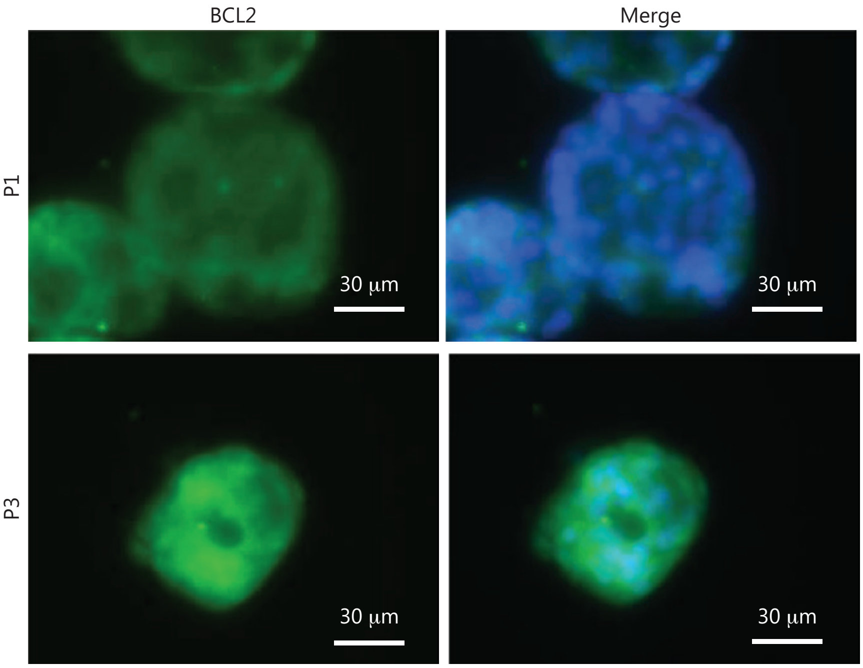

Figure S5 Immunofluorescence assay of BCL2 for GN4 organoids from P0 and P3, showing increased protein expression of BCL2.

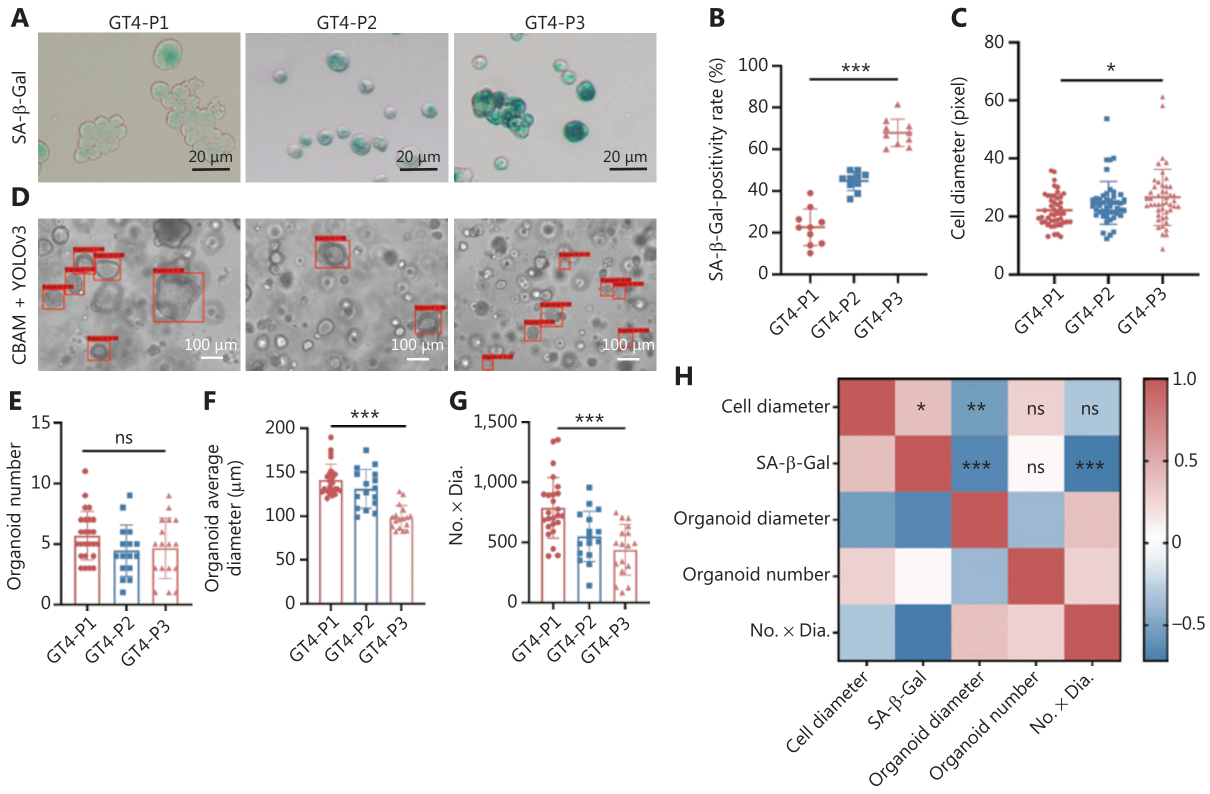

Figure S6 Senescence features of the GT4 organoid from gastric cancer. (A) Increased staining intensity of SA-β-Gal with extended passaging from P1 to P3. (B) Increased total SA-β-Gal-positivity rate from P1 to P3 (***P < 0.0001). (C) Gradually increased single cell diameter of GT4 organoids from P1 to P3 (*P = 0.0289). (D) Evaluative outputs of the CBAM-YOLOv3 model from P1 to P3. (E) The organoid numbers are not clearly changed (^{ns}P = 0.154). (F) Gradually decreased organoid average diameter (***P < 0.0001). (G) Decreased No. × Dia. from P1 to P3 (***P < 0.0001). (H) Correlation analysis of observation parameters of SA-β-Gal staining and single-cell diameter with evaluative parameters by using the CBAM-YOLOv3 model. The red box represents positive correlation, and the blue box represents negative correlation. The stronger the coefficients, the darker the color. The single-cell diameter positively correlates with SA-β-Gal-positivity rate (Pearson r = 0.3826, *P = 0.0369). No correlation is observed between the SA-β-Gal-positivity rate and organoid number (Pearson r = 0.0567, ^{ns}P = 0.7658). The positivity rate of SA-β-Gal staining negatively correlates with the organoid average diameter (Pearson r = −0.6596, ***P < 0.0001) and No. × Dia. (Pearson r = −0.7187, ***P < 0.0001). No significant correlation is observed between the single-cell diameter and organoid number (Pearson r = −0.2872, ^{ns}P = 0.1239) and No. × Dia. (Pearson r = −0.3151, ^{ns}P = 0.0899). The single-cell diameter is negatively associated with the organoid average diameter (Pearson r = −0.5365, **P = 0.0022). n = 3, passage number. The experiments were repeated 3 times, and results are shown as mean ± SD.

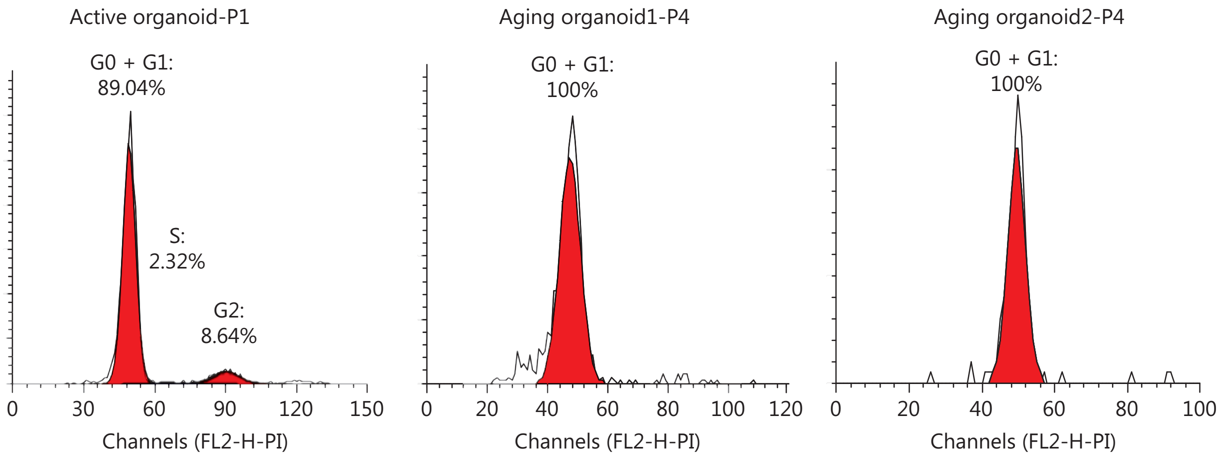

Figure S7 Cell cycle assay for one case from an active organoid (P1) and 2 cases from aging organoids (P4). The active organoid shows fewer G0/G1 cells (89.04%), than the 2 aging organoids (100% and 100%, respectively).

Table S1 Primers for qRT-PCR

Primers for qRT-PCR	Company
Forward: *p21*<br>AGTCAGTTCCTTGTGGAGCC	Sangon Biotech
Reverse: *p21*<br>CATTAGCGCATCACAGTCGC	Sangon Biotech
Forward: *p15*<br>TTTACGGCCAACGGTGGATT	Sangon Biotech
Reverse: *p15*<br>CATCATCATGACCTGGATCGC	Sangon Biotech
Forward: *p16*<br>GGGTCGGGTAGAGGAGGTG	Sangon Biotech
Reverse: *p16*<br>GCTGCCCATCATCATGACCT	Sangon Biotech
Forward: *CCNA2*<br>TGGTGGTCTGTGTTCTGTGAA	Sangon Biotech
Reverse: *CCNA2*<br>CTTGGATGCCAGTCTTACTCA	Sangon Biotech
Forward: *CCNE2*<br>TAGCTGGTCTGGCGAGGTTT	Sangon Biotech
Reverse: *CCNE2*<br>ACAGGTGGCCAACAATTCCT	Sangon Biotech
Forward: *LMNB1*<br>GCGTGCGTGTCTATGCTAAG	Sangon Biotech
Reverse: *LMNB1*<br>GCTTCCAACTGGGCAATCTG	Sangon Biotech

Table S2 Clinicopathological information on organoids used for aging evaluation

Cases	Gender	Age, years	Location	Borrmann	Histology	Passages
Tumor						
Active organoids						
GT1	Female	65	Corpus	I	Adenocarcinoma	P45
2003GT6-1	Female	68	Antrum	III	Adenocarcinoma	P41
GT6-2	Female	68	Antrum	III	Adenocarcinoma	P42
GT20-2	Male	50	Antrum	II	Adenocarcinoma	P30
GT28	Female	39	Corpus and antrum	IV	Signet-ring cell carcinoma	P28
GT29	Male	77	Corpus and antrum	III	Adenocarcinoma	P25
Senescent organoids						
GT3	Male	65	Antrum	III	Adenocarcinoma	P6
GT4	Male	72	Cardia	III	Adenocarcinoma	P6
GT14	Male	74	Cardia	III	Adenocarcinoma	P5
GT15	Female	45	Antrum	III	Signet-ring cell carcinoma	P5
Normal						
GN2	Female	33	Corpus and antrum	IV	/*	P7
GN3	Male	65	Antrum	III	/	P5
GN4-1	Male	72	Cardia	III	/	P5
GN4-2	Male	72	Cardia	III	/	P5
GN5	Female	54	Corpus and antrum	III	/	P7
GN6	Female	68	Antrum	III	/	P6
GN8	Male	66	Corpus and antrum	IV	/	P4
GN9	Female	62	Corpus and antrum	III	/	P5
GN17	Male	74	Angle	III	/	P5

*/ represents the corresponding normal histology from the stomach.
